# Increasing incidence of invasive group A streptococcal (iGAS) outbreaks in England 2015–2019

**DOI:** 10.1017/S0950268825000226

**Published:** 2025-03-12

**Authors:** Iona Smith, Elizabeth Marchant, Juliana Coelho, Megan Bardsley, Amanda Wright, Nicola Love, Sooria Balasegaram, Colin S Brown, Dan Todkill, Theresa Lamagni, Valérie Decraene

**Affiliations:** 1 UK Field Epidemiology Training Programme, UK Health Security Agency, London, UK; 2 UK Health Security Agency, London, UK; 3 Public Health Wales, Cardiff, Wales

**Keywords:** England, invasive group A streptococcal outbreaks, iGAS outbreaks, surveillance

## Abstract

Early detection and active management of invasive group A *Streptococcus* (iGAS) infection outbreaks are essential. Here, we describe the changing epidemiology of outbreaks of iGAS in England between 2015 and 2019, a period of increasing incidence of iGAS infection. Data on iGAS infections were extracted from national public health management records and laboratory records. Outbreaks were described in size, duration, setting, and *emm* type. Overall, 194 outbreaks were identified, and reports increased each year, from 16 outbreaks in 2015 to 61 in 2019. The median outbreak size was 3 cases (*n* = 37; 19%), with 27% of outbreaks recording 4–10 cases (*n* = 53) and 7% recording more than 10 cases (*n* = 13). Outbreak duration ranged from 0 to 170 weeks (median 7). Settings of outbreaks changed over the study period, with increasing numbers observed in multiple settings. This study provides new insights into the changing burden of iGAS infection and outbreaks in England.

## Introduction

The bacterium *Streptococcus pyogenes*, more commonly referred to as group A *Streptococcus* (GAS), causes many different conditions, ranging from mild illnesses in the throat and skin to severe life-threatening invasive disease (iGAS) [1]. Most cases of GAS are sporadic, but outbreaks occur in a range of community and healthcare settings and are often challenging to control [2, 3].

Direct person-to-person transmission can occur through inhalation of respiratory particles or through skin contact, with contamination of the environment also playing a role [4]. GAS is primarily a community-acquired pathogen with an estimated asymptomatic carriage of <1% [5]. Those at higher risk of developing iGAS infections include children with viral infections (varicella and influenza), peripartum women, the elderly, and individuals with skin lesions or who are immunocompromised [6]. iGAS infections occur when GAS bacteria invade a normally sterile body site, for example, blood and cerebrospinal fluid. iGAS infection has a mortality rate of 8–16% [1, 2], and clinical manifestations of iGAS disease include necrotizing fasciitis, streptococcal toxic shock syndrome, pneumonia, septicaemia, and meningitis [1, 2].

While GAS can cause severe infections, cases of iGAS are uncommon, with rates typically between 3 and 5 per 100,000 population in England [5]. In England, iGAS infections are notifiable, and outbreaks are detected through established iGAS surveillance, supplemented by microbial characterization [7]. Furthermore, all iGAS isolates should be referred for typing at the UK Health Security Agency (UKHSA) *Staphylococcus* and *Streptococcus* reference laboratory, as this is the primary mechanism for outbreak detection. Microbial characterization of *S. pyogenes*, by its M protein (encoded by the *emm* gene) [6], is used to assess the relatedness of strains, with over 200 *emm* types documented to date [8, 9]. Increasingly, whole genome sequencing (WGS) has been used to provide further discrimination, aiding outbreak investigation [10–12].

In England since 2018, there have been an increasing number of iGAS outbreaks associated with home healthcare, such as community nursing [2, 13]. Several outbreaks among people who inject drugs (PWID) have also been reported [14–17]. However, few systematic assessments of iGAS outbreaks have been conducted, undermining our ability to learn about how and where outbreaks occur. This paper uses routinely collected national surveillance data to describe the epidemiology of outbreaks of iGAS in England between 2015 and 2019. We contribute to the scientific evidence base by summarizing the epidemiology of outbreaks of iGAS in terms of size, setting type, duration, and *emm* type and use modeling to determine whether the size and duration of outbreaks changed over time.

## Methods

### Microbiological characterization

As part of national surveillance and to support outbreak investigations, invasive and non-invasive GAS cases were referred to the UKHSA reference laboratory for typing. Bacterial strains were cultured using standard methods [18], and *emm* gene sequence typing was undertaken as previously described [19–21].

### Data collection

Data on outbreaks of iGAS (as defined below) notified between 1st January 2015 and 31st December 2019 in England were collated and extracted from the national electronic case management system used by regional Health Protection Teams to log all incidents and outbreaks (HPZone) and supplemented with information held at the reference laboratory. Information extracted included outbreak start and end dates, setting (e.g. care home, prison/custodial, etc.), and the number of confirmed cases and *emm* type, where available. Laboratory-confirmed cases of iGAS were extracted from the Second Generation Surveillance System (SGSS), which collates data on microbiological diagnoses made by laboratories across England. The data used for this study can be requested from UKHSA, and these requests will be considered.

## Definitions

### Confirmed iGAS case

An individual with isolation of GAS from a normally sterile body site, such as blood, cerebrospinal fluid, joint aspirate, pericardial/peritoneal/pleural fluids, bone, endometrium, deep tissue, or deep abscess at operation or post-mortem. This also included severe GAS infections where GAS had been isolated from a normally non-sterile site in combination with a severe clinical presentation, such as streptococcal toxic shock syndrome or necrotizing fasciitis.

### Probable iGAS case

An individual who has a severe clinical presentation consistent with iGAS infection, in the absence of microbiological confirmation of GAS, and either the clinician considers that GAS is the most likely cause or there is an epidemiological link to a confirmed case.

### Outbreak

For this study, an outbreak was defined as two or more confirmed cases of iGAS identified by the Health Protection Team as being linked by person, place, and time and which were recorded as a cluster, outbreak, or issue. The duration of an outbreak was calculated as the interval between the date of onset of the first and last case, where data was available. Non-invasive cases of GAS were excluded due to non-systematic recording in HPZone. The setting of an outbreak was selected from one of the following: care home, community, community nursing, homeless/hostel/shelter, hospital/maternity, household, other, prison/custodial, or school/nursery/university.

## Data analysis

Laboratory-confirmed cases of iGAS were extracted from SGSS, and rates were calculated using mid-year resident population estimates produced by the Office for National Statistics [22]. To calculate the total number of sporadic cases of iGAS reported in England each year, confirmed cases of iGAS associated with an outbreak were removed from the annual total of iGAS cases reported through SGSS.

Further statistical analyses were conducted to assess whether the size and duration of outbreaks changed over time. To investigate the relationship between time (year), size of an iGAS outbreak (measured in number of confirmed cases), and duration of an outbreak (measured in days), negative binomial regression models were used. Duration data was transformed using the Haldane-Anscombe correction, which lead to 0.5 days being added to all outbreak duration values to allow six outbreaks with zero days in length to be included in the statistical analyses. Year and setting of outbreaks were included in negative binomial regression models investigating changes in size and duration of outbreaks. Models were developed, including year as a continuous variable, due to investigating the trend over the five-year period. Sensitivity analyses were conducted with year as a categorical variable, and in these models, all coefficient confidence intervals overlapped. All statistical analyses were conducted in R and R studio (version: 4.3.1).

## Results

**Figure 1.**
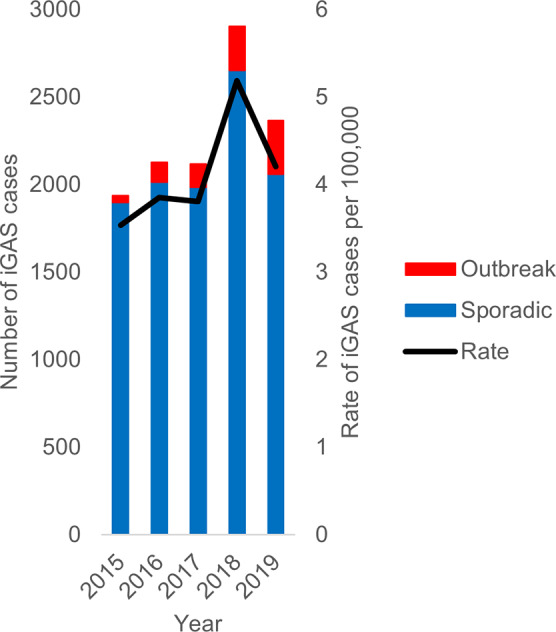
Number of cases of iGAS and rate per 100,000 in England between 2015 and 2019.

**Figure 2.**
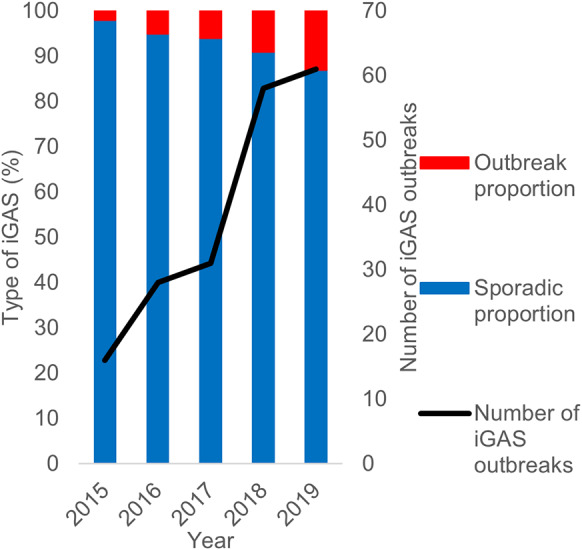
Proportion of outbreak and sporadic iGAS cases and number of iGAS outbreaks in England between 2015 and 2019.

Between 2015 and 2019, iGAS cases increased from 1938 to 2368 in England (Figure 1), an increase in rate from 3.5 to 4.2 cases per 100,000 population. During this five-year period, 194 outbreaks were identified with a corresponding 846 outbreak cases reported. The annual number of outbreaks increased more than threefold from 16 in 2015 to 61 in 2019. Sporadic cases contributed more than 90% of total cases in each year between 2015 and 2018 (overall 94%, 8551/9090), while in 2019, 87% of iGAS cases were sporadic (Figure 2).

### Size of outbreaks

The median number of confirmed cases per outbreak was 2–3 cases each year, and the range was 2–36 over the study period (Table 1). The total number of confirmed cases within an outbreak increased between 2015 and 2019, with a maximum of 36 cases reported from a 2018 outbreak (Table 1). However, we did not detect a significant relationship between year and number of confirmed cases (*n* = 194 outbreaks; IRR: 1.1 95% CI 1.0–1.2 p = 0.2; Table 2), having adjusted for setting type.

**Table 1.** Size and duration of iGAS outbreaks between 2015 and 2019 in England

Year	Total number of outbreaks	Number of confirmed cases in outbreak (*n* = 194)		Duration of outbreak (weeks; *n* = 169)	
		Median	Minimum–maximum	Median	Minimum–maximum
2015	16	2	2–6	3	1–20
2016	28	3	2–14	10	0–71
2017	31	2	2–21	13	0–100
2018	58	3	2–36	5	0–170
2019	61	3	2–33	7	0–71
Total	194	3	2–36	7	0–170

**Table 2.** Factors associated with iGAS outbreak size (number of confirmed cases) from 2015 to 2019 in England (*n* = 194)

	Univariable			Multivariable		
Characteristic	IRR	95% CI	*P-*value	IRR	95% CI	*P-*value
Year	1.1	1.0–1.2	0.01	1.1	1.0–1.2	0.2
Setting						
Household	Ref	Ref	Ref	Ref	Ref	Ref
Homeless/hostel/shelter	2.6	1.7–4	<0.01	2.6	1.7–4.1	<0.0001
Community nursing	2.4	1.5–3.9	<0.01	2.3	1.4–3.8	<0.001
Community	2.2	1.4–3.6	<0.01	2.2	1.4–3.5	<0.001
Other	1.4	0.5–4.2	0.5	1.4	0.5–4.1	0.6
Care home	1.1	0.7–1.7	0.7	1.1	0.7–1.7	0.6
Hospital/maternity	1.1	0.7–1.7	0.7	1.1	0.7–1.8	0.5
Prison/custodial	0.9	0.4–2.1	0.8	0.9	0.3–2.1	0.7
School/nursery/university	0.7	0.2–2.4	0.6	0.7	0.2–2.6	0.6

### Duration of outbreaks

Of the 194 iGAS outbreaks included in our analysis, 25 did not have duration data available. Of the remainder (*n* = 169), the median outbreak duration changed each year, with a median outbreak length of 7 weeks over the study period (Table 1). While the longest iGAS outbreaks, at 100 and 170 weeks, respectively, were reported in 2017 and 2018, there was no evidence to suggest a significant trend in the duration of outbreaks over the study period, adjusting for setting type (*n* = 169 outbreaks; IRR: 1.0 95% CI 0.8–1.1 *p* = 0.7; Table 3).

**Table 3.** Factors associated with iGAS outbreak duration (in days) from 2015 to 2019 in England (*n* = 169)

	Univariable			Multivariable		
Characteristic	IRR	95% CI	*P-*value	IRR	95% CI	*P-*value
Year	1.1	0.9–1.3	0.4	1	0.8–1.1	0.7
Setting						
Household	Ref	Ref	Ref	Ref	Ref	Ref
Homeless/hostel/shelter	4.5	2.1–9.4	<0.01	4.4	2.0–9.3	<0.0001
Community nursing	2.7	1.1–7.3	0.04	2.7	1.1–7.2	0.04
Community	2.3	0.9–5.5	0.1	2.3	0.9–5.5	0.06
Other	1.9	0.4–19.9	0.5	1.9	0.4–20.3	0.5
Hospital/maternity	1.2	0.5–2.3	0.7	1.1	0.5–2.3	0.7
Care home	1.1	0.5–2.1	0.8	1	0.5–2.1	0.9
School/nursery/university	0.6	0.1–6.4	0.6	0.6	0.1–6.4	0.6
Prison/custodial	0.3	0.1–1.3	0.1	0.3	0.1–1.3	0.05

### Outbreak setting

**Figure 3.**
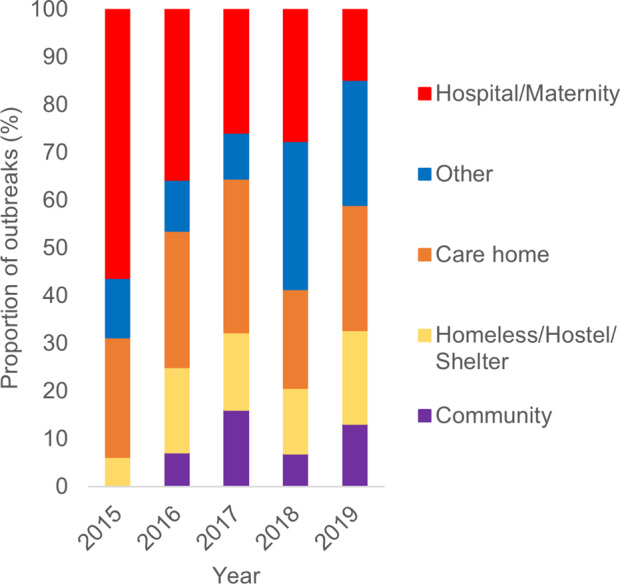
Proportion of iGAS outbreaks in England by contextual setting and year, 2015–2019 (*n* = 194).

The most common outbreak settings were in hospitals, including maternity units (27%; *n* = 52), followed by care homes (26%; *n* = 50) and homeless shelters (16%; *n* = 31) (Table 4). An increase in the number of outbreaks between 2015 and 2019 was seen in most settings. Hospital/maternity units, however, accounted for a decreasing proportion of iGAS outbreaks each year during the study period, reducing from 56% of outbreaks in 2015 to 15% in 2019 (Figure 2), although the number of outbreaks reported each year remained fairly consistent (between 9 and 16; Table 4). The frequency of outbreaks increased, notably in care homes (from 4 to 16), homeless shelters (1–12), and community nursing (0–6). These three settings accounted for 56% of outbreaks in 2019 compared to 31% in 2015 (Figure 3). The longest outbreaks were observed in homeless shelters (duration 0–170 weeks; median 38; Table 5), followed by care homes (duration 0–71 weeks; median 6; Table 5) and household settings (duration 0–70 weeks; median 1; Table 5). It was observed that, adjusting for year of outbreak, iGAS outbreaks reported in homeless shelters and community nursing were of significantly longer duration than those in household settings (IRR: 4.4 95% CI 2.0–9.3 *p* = <0.01 and IRR: 2.7 95% CI 1.1–7.2 *p* = 0.04, respectively; Table 3). The largest outbreaks were observed in community settings (2–36 cases; median 3), defined as cases occurring within the community and not within another specified setting, followed by community nursing (2–33 cases; median 4) and homeless shelter settings (2–28 cases; median 5; Table 5). Outbreaks observed in all three settings were found to be significantly larger compared to household settings, adjusting for year of outbreak (IRR: 2.3 95% CI 1.4–3.8 *p* = <0.001, IRR: 2.3 95% CI 1.4–3.8 *p* = <0.001, and IRR: 2.6 95% CI 1.7–4.1 *p* = <0.0001, respectively; Table 2).

**Table 4.** Number of iGAS outbreaks by setting and by year in England, 2015–2019

Contextual setting	2015		2016		2017		2018		2019		Total	
	No.	%	No.	%	No.	%	No.	%	No.	%	No.	%
Care home	4	25	8	29	10	32	12	21	16	26	50	26
Community	0	0	2	7	5	16	4	7	8	13	19	10
Community nursing	0	0	1	4	3	10	6	10	6	10	16	8
Homeless shelters	1	6	5	18	5	16	8	14	12	20	31	16
Hospital/maternity	9	56	10	36	8	26	16	28	9	15	52	27
Household	1	6	2	7	0	0	9	16	6	10	18	9
Prison/custodial	0	0	0	0	0	0	2	3	2	3	4	2
School/nursery/university	1	6	0	0	0	0	0	0	1	2	2	1
Other	0	0	0	0	0	0	1	2	1	2	2	1
Total	16	100	28	100	31	100	58	100	61	100	194	100

**Table 5.** Duration and size of iGAS outbreaks by setting in England between 2015 and 2019

Contextual setting	Duration (*n* = weeks)			Size (*n* = cases)		
	Min	Max	Median	Min	Max	Median
Care home	0	71	6	2	18	2
Community	0	64	7	2	36	3
Community nursing	1	56	17	2	33	4
Homeless/hostel/shelter	0	170	38	2	28	5
Hospital/maternity	0	53	4	2	8	2
Household	0	70	1	2	6	2
Prison/custodial	0	6	1	2	3	3
School/nursery/university	3	7	5	2	2	2
Other	7	26	16	2	6	4
Grand total	0	170	7	2	36	3

### Novel emm type emergence

The most common *emm* types were *emm* 89.0, 1.0, and 66.0, accounting for 13%, 12%, and 9% of the outbreaks, respectively (Table 6). *Emm* 108.1 emerged in 2017 and was predominantly reported in outbreaks in homeless shelters. However, *emm* typing was not available for every outbreak; 12% of iGAS outbreaks had no *emm* type recorded.

**Table 6.** Number and proportion of outbreaks by *emm* type^a^ split and contextual setting for iGAS outbreaks in England between 2015 and 2019

*emm* type	Care home		Community		Community nursing		Homeless shelter		Hospital/maternity		Household		Prison		Education		Other		Total	
	No.	%	No.	%	No.	%	No.	%	No.	%	No.	%	No.	%	No.	%	No.	%	No.	%
89	9	17	4	14	5	29	5	7	10	13	0	0	1	25	0	0	0	0	34	13
1	11	21	0	0	2	12	2	3	15	19	3	16	0	0	0	0	0	0	33	12
66	2	4	3	11	0	0	17	25	2	3	0	0	0	0	1	20	0	0	25	9
108.1	2	4	4	14	0	0	8	12	2	3	0	0	0	0	2	40	0	0	18	7
28	5	9	2	7	1	6	2	3	6	8	1	5	0	0	0	0	0	0	17	6
94	5	9	2	7	2	12	5	7	2	3	1	5	0	0	0	0	0	0	17	6
11	5	9	0	0	0	0	3	4	1	1	1	5	0	0	1	20	0	0	11	4
87	4	8	1	4	1	6	2	3	3	4	0	0	0	0	0	0	0	0	11	4
82	0	0	2	7	0	0	4	6	2	3	1	5	0	0	0	0	0	0	9	3
5.23	1	2	0	0	0	0	0	0	3	4	3	16	0	0	0	0	0	0	7	3
3.93	0	0	0	0	0	0	0	0	4	5	1	5	1	25	0	0	0	0	6	2
4	2	4	0	0	0	0	1	1	1	1	1	5	0	0	0	0	1	50	6	2
12	0	0	0	0	1	6	0	0	2	3	3	16	0	0	0	0	0	0	6	2
77	1	2	1	4	0	0	2	3	2	3	0	0	0	0	0	0	0	0	6	2
81	1	2	1	4	0	0	3	4	0	0	0	0	0	0	0	0	0	0	5	2
Total	53		28		17		67		77		19		4		5		2		272	

a Some outbreaks may have multiple *emm* type.

## Discussion

Our review included over 190 outbreaks of iGAS infection. The high and increasing number of outbreaks highlights the public health burden of these infections and the associated impact on communities and the healthcare economy. Between 2015 and 2019, the number of iGAS cases and outbreaks increased, with a peak of cases reported in 2018 [13, 23]. The reason for the increase in both remains unclear, but the proportion of sporadic iGAS cases remained reasonably constant over this period, except for 2019, which saw a greater proportion of cases associated with outbreaks compared to previous years. During the study period, there were no changes to diagnostic testing or the number of reporting healthcare facilities/laboratories that would have impacted the identification of iGAS cases through SGSS. The increase in iGAS cases associated with outbreaks could, however, be due to improved investigation of individual cases, resulting in the identification of epidemiological links and identification of outbreaks. It could also be due to improved recording of common exposures and settings. This would explain why the number of outbreaks recorded tripled over this five-year period, whereas the number of recorded cases only rose by 8%, noting the recording mechanisms on HPZone did not change during this time period. Additionally, there were no notable changes at the national level in terms of disease surveillance and outbreak investigation, which would account for the increase in outbreaks detected.

Interestingly, the proportion of iGAS outbreaks identified in hospital settings decreased over the study period, despite the overall increase of iGAS outbreaks observed. The increase of outbreaks in homeless shelters and community nursing reflects the findings by Nabarro and Valenciano [2, 24]. This could indicate a true increase of iGAS outbreaks in these settings, an improvement in detection, or a combination of both these factors, and warrants further investigation. Given that there was an increased understanding by public health teams about iGAS, it is likely some of this is due to increased ascertainment of other outbreak settings, particularly in care homes, community nursing, and homeless shelters. Using rough sleeping as a proxy for those who use homeless shelters, the estimated number of people rough sleeping increased between 2015 and 2019 (n = 3569 and 4266, respectively; number of people rough sleeping on a single night in autumn in England), with a peak number of rough sleepers recorded in 2017 [25]. This could suggest that the number of those using homeless shelters has increased, therefore increasing the risk of iGAS outbreaks in these settings. Additionally, logistical challenges of applying control measures in these settings may have contributed to their size and/or length. There are challenges to ensuring implementation and compliance of antibiotic chemoprophylaxis in homeless shelters due to the transient nature of the accommodation they provide [5, 10, 16, 26].

It is unclear what is driving the increase in iGAS outbreaks in community nursing settings but given England’s ageing population and utilization of community nursing, greater awareness of outbreaks in these settings is critical [2]. Improvements in detection and in iGAS case management may provide an alternative explanation for the changes observed in the settings reporting iGAS outbreaks. Published guidance for preventing and controlling iGAS infections in healthcare and maternity settings was introduced in 2012, which may have contributed to the relative reduction in the proportion of outbreaks in these settings [27].

It is possible that undetected outbreaks occurred that were not included in this study, and those outbreaks not captured may differ substantially to the outbreaks presented here. It is plausible that any unreported outbreaks may have occurred within the community in underserved populations with limited access to healthcare, leading to cases being undetected [28]. It is also possible that some outbreaks extended into 2020 and were not included in our analysis, as if an outbreak did not have an end date, it was excluded from the analysis. Due to our inclusion of outbreaks comprising two or more confirmed cases, we are certain to have missed outbreaks comprising one confirmed and one probable case. However, at the time, guidelines did not have a probable case definition, so the data may have been recorded differently and in a non-systematic manner. Another limitation is the potential impact of the different ways regional health protection teams use and record information on HPZone. For example, the outbreak setting could be categorized differently if the case concerned multiple settings, i.e. a case who attended school and was subsequently hospitalized.

When investigating the relationship between year, duration, and size of outbreaks, we were aware that different settings were likely to report different numbers of confirmed cases due to the ability to rapidly detect cases [2] and successfully implement control measures, and so we adjusted for setting within both final models. We did not find evidence to suggest that outbreaks were getting larger or smaller, or longer or shorter, suggesting that outbreak size and duration have remained consistent overall, with specific variation between settings. The faster an outbreak is detected, the quicker control measures can be implemented, which will result in shorter and smaller outbreaks.

We found that the highest number of outbreaks were reported from hospital maternity units and care homes. This likely reflects the well-documented vulnerability of individuals engaged with and living in these settings [26, 29]. Care homes are vulnerable to outbreaks of iGAS in part due to degradation of skin integrity among the elderly population that they serve. This may enable carriage of GAS and result in this population acting as reservoirs for infection, with subsequent seeding of outbreaks requiring awareness of this risk and appropriate actions being taken, for example, testing for carriage of GAS in care home settings [30, 31]. Updated guidelines for the management of contacts of iGAS cases in community settings, which provide recommendations and guidance for new target groups (for example, late-stage pregnant women and the elderly) for antibiotics, could help in reducing iGAS outbreaks among these settings [5]. Further research is necessary to assess the impact of this guidance on iGAS outbreaks in community settings.

The duration of outbreaks differed substantially between settings. This is partly due to previously highlighted challenges in both outbreak detection/management in difficult settings with underserved populations and the ability to detect, link, and manage cases. This study detected *emm* types 89.0 and 1.0 most frequently in a range of settings. This reflects others’ findings, with a previous study in 2014 reporting e*mm* type 1.0 and 89.0 accounting for >5% of infections among the study population [32]. *Emm* type 108.1 emerged in 2017, predominantly associated with outbreaks in homeless shelters. It is possible that certain *emm* types are more likely to cause outbreaks, which could be reflected in the *emm* types found in this study, but this is difficult to prove. While *emm* typing is useful to distinguish GAS strains, the application of WGS in outbreak settings has a higher discriminatory power and allows for the correct inclusion/exclusion of epidemiologically linked cases, which may occur over a long period [3]. In the future, universal implementation of WGS for all sporadic iGAS cases would also allow us to more accurately classify seemingly sporadic cases as belonging to an outbreak; this is particularly useful for outbreaks extending over a long period of time and/or a wider geographic area [3, 28].

This study aimed to improve the understanding around the changing epidemiology of iGAS outbreaks in England over a five-year period and provides a useful baseline for future comparison. During this time, a marked increase in iGAS outbreak incidence was observed, with outbreaks of iGAS detected across diverse populations in a range of settings. Specifically, there was an increase in outbreaks in care home settings, household settings, in the community, and among those who receive community nursing and those who use homeless shelters. The increasing number of outbreaks highlights the continued need for prompt public health management, especially given the complex and dispersed populations affected. Routine adoption of WGS during an iGAS outbreak could help facilitate the early identification of outbreaks. There is also an opportunity with data from subsequent years to analyze the impact of newly published community guidelines for the management of contacts of iGAS infection in community settings in England [5] and assess if this leads to a reduction in the number, size, and duration of iGAS outbreaks observed within the community. This analysis would also allow for the description and investigation of any impact that the COVID-19 pandemic may have had on the epidemiology of iGAS outbreaks in England. While the lifting of pandemic restrictions has been linked to a surge in iGAS infections across Europe [33], including within England [34], changes in outbreak settings have not been systematically assessed.
